# Empowering healthcare professionals to help smokers quit: Relevance of a smoking cessation online training program

**DOI:** 10.1016/j.puhip.2025.100699

**Published:** 2025-12-11

**Authors:** Dalia Alleaume, Yasmine Célia Benrabah, Ingrid Allagbé, Marie Masure, Anneliese Depoux, Marie Malécot, Anne-Laurence Le Faou

**Affiliations:** a Association Robert Debré pour la Recherche Médicale, Paris, France; b Société Francophone de Tabacologie, Paris, France; c Centre des Politiques de la Terre, Université Paris Cité, Paris, France; d Outpatient Addictology Center, Georges Pompidou European Hospital, AP-HP Centre- Université Paris Cité, Paris, France

**Keywords:** MOOC, Smoking cessation, Education, Healthcare professionals, Satisfaction, Success rate

## Abstract

**Objectives:**

Healthcare professionals are instrumental in aiding smokers to quit, necessitating evidence-based smoking cessation education. Massive Open Online Courses (MOOCs) provide a flexible platform for such education. This study assessed the satisfaction of health professionals with the content of the MOOC “Smoking: Quit Your Own Way!”, distributed over the period 2020–2023, and their success rates in completing of this course.

**Study design:**

We conducted a descriptive study.

**Methods:**

The study based on quantitative data from 4229 learners on France Université Numérique (FUN) and 1488 on Pédagogie Numérique en Santé (PNS) platforms who completed the satisfaction questionnaire on the content of the MOOC and who answered the quizzes offered during the course.

**Results:**

Most participants were women (FUN: 79.7 %; PNS: 84.0 %), with nurses being the largest group (FUN: 27.7 %; PNS: 28.6 %). Satisfaction was high (97.9 %). Weekly quiz success rates ranged from 15.5 % to 30.0 %. Rehabilitation professionals had the highest success rate at 40.2 %, followed by medical doctors at 35.7 %, and dental surgeons at 34.9 %. Midwives and nurses both had a success rate of 32.2 %. Other notable rates included prevention professionals at 30.9 % and psychologists at 27.6 %. The lowest success rates were seen in social professions (16.0 %) and nurse assistants (9.4 %).

**Conclusions:**

The “Smoking: Quit Your Own Way!” MOOC effectively train healthcare professionals in smoking cessation, enhancing their theoretical and practical skills to support smokers.

## 1 Introduction

In France, cigarette consumption remains high, with 24.5 % of the population smoking daily [1]. Given the impact of tobacco smoking on health burden, it is crucial to facilitate measures promoting smoking cessation (SC). Yet, providing SC support for individuals is essential for public health, with evidence-based methods being taught to healthcare professionals to help them encourage smokers to quit [2].

To improve smokers' access to SC pharmacotherapies, in 2016, French health authorities expanded the authorization of nicotine replacement therapy (NRT) prescription to nurses, physiotherapists, and dentists. Previously, NRT prescriptions were limited to medical doctors and midwives. Nurses, as the largest group of healthcare professionals, are particularly vital in tobacco control due to their frequent patient interactions. Hence, tailored SC training for nurses is valuable, as nursing SC interventions have been shown to increase 6-month cessation rates by 30 % [3].

Recognizing the importance of offering SC training to healthcare professionals, a Massive Open Online Course (MOOC), entitled “Smoking: Quit Your Own Way!”, was disseminated in 2020–2023 [4]. MOOCs represent a cutting-edge trend in higher education, conceptualized as open-access, global, and free online platforms providing video-based instructional content [5]. The MOOC “Smoking: Quit Your Own Way!” delivers scientific and practical information on engaging smokers in SC and provides training materials for conducting routine cessation visits and group sessions. The purpose of this study was to evaluate the sociodemographic characteristics of participants registered in the MOOC “Smoking: Quit Your Own Way!” and assess their satisfaction level and success rates in completing the MOOC.

## 2 Methods

### 2.1 MOOC description

The MOOC “Smoking: Quit Your Own Way!” covers various aspects of tobacco use and cessation support. Detailed content for each module is provided in Table S1. The MOOC content is mainly available in French, otherwise in English with French subtitles. The course spans seven weeks' modules, with each module requiring 2 h of participation. Each week module concludes with a summary of the content and animations to aid memorization. A discussion forum allows participants to share their experiences as well as to ask questions to the MOOC team. Overall, the MOOC includes 49 videos including practical workshops and quizzes corresponding to each week module's contents. Each module contains between 10 and 14 quizzes. MOOC participants were deemed successful if they answered at least half of the quiz questions correctly, qualifying them to receive a MOOC certificate of success. Participants had the option to register for the MOOC on two platforms during the study period: France Université Numérique (FUN) and Pédagogie Numérique en Santé (PNS). The primary objective of the FUN platform is to provide online education on various academic fields, while the PNS platform is dedicated to healthcare education. Additional information is available in **Supplemental materials**.

### 2.2 Data collection

On the FUN platform, data were collected over four sessions from 2020 to 2021, while on the PNS platform, it spanned from 2021 to 2023. Sociodemographic information was extracted for each participant, including gender, age, country of residence, educational level, and occupation. The country of residence was categorized into France, French-speaking countries, and non-French-speaking countries. At the end of the MOOC, participants voluntarily completed a satisfaction questionnaire, assessing their satisfaction with the course using a 5-point Likert scale, ranging from “very unsatisfied” to “unsatisfied”, “neutral”, “satisfied”, and “very satisfied”. Participation rates in the weekly quizzes and attainment of certificates of success were also examined on both platforms.

### 2.3 Data analysis

Results were presented as numbers and percentages for categorical variables, and as median and range for continuous variables. Associations between MOOC success and types of professionals were assessed using logistic regression. Missing data were not imputed. Data were analyzed using R.

## 3 Results

On the FUN platform, 4229 learners answered the MOOC “Smoking: Quit Your Own Way!” questionnaires from 2020 to 2021 while on the PNS platform, 1488 learners completed them in 2021–2023. Participants on the FUN platform shared similar sociodemographic characteristics to those on the PNS platform (Table S2). On the FUN platform, participants had a median age of 34 years, with 79.7 % being women and 87.3 % residing in France. Additionally, 81.6 % had completed postgraduate studies. Likewise, on the PNS platform, the median age of participants was 36 years, with 84.0 % being women. Most of the PNS participants lived in France (88.9 %) and had completed postgraduate studies (92.2 %). Overall, more than half of the learners (57.5 % on FUN and 69.4 % on PNS) were employed in the healthcare sector, with nurses being predominant among participants on both platforms (27.7 % on FUN and 28.6 % on PNS). In total, 97.9 % of participants on both platforms responded that they were satisfied or very satisfied with the MOOC.

Fig. S1 investigates the quiz success rates according to the weekly modules and the platforms. The overall success rate of the MOOC was 15.9 % on FUN and 19.3 % on PNS.

**Fig. 1:**
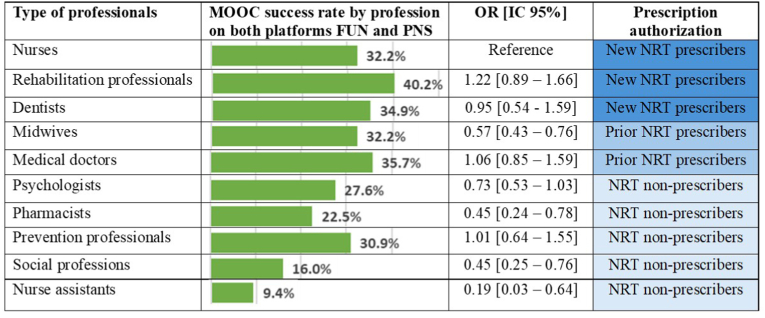
Massive Open Online Course Success Rates (France Université Numérique and Pédagogie Numérique en Santé) by Profession: Assessment of Associations using logistic regression.

Fig. 1 illustrates MOOC success rates by profession, combining data from both the FUN and PNS platforms. Rehabilitation professionals had the highest success rate at 40.2 %, followed by medical doctors at 35.7 %, and dentists at 34.9 %. Midwives and nurses both had a success rate of 32.2 %. Other notable rates included prevention professionals at 30.9 % and psychologists at 27.6 %. The lowest success rates were seen in social professions (16.0 %) and nurse assistants (9.4 %). Using nurses as the reference group, midwives, pharmacists, social professionals, and nurse assistants had significantly lower odds of successfully completing the MOOC. No significant differences were observed for rehabilitation professionals, dentists, medical doctors, psychologists and prevention professionals (Fig. 1).

When excluding non-responders, quiz success rates during the 7-week MOOC on both platforms are shown in Fig. S2. Consistent quiz performance was observed throughout the MOOC duration on both platforms with health professionals succeeding in the first five weeks, while prevention professionals excelled particularly in the public health modules (weeks 6 and 7).

## 4 Discussion

The purpose of this study was to evaluate the adoption of an evidence-based MOOC content entitled “Smoking Cessation: Quit Your way!” and its impact on participants. The study findings revealed a high satisfaction rate with the MOOC, exceeding 95 %, reflecting strong approval of its content and delivery.

Our study revealed a diverse participant profile. Among the participants, 28 % were nurses. The higher participation level of nurses in our study, along with their active engagement in acquiring knowledge in SC strategies, could be attributed to the 2016 authorization for nurses in France to prescribe NRT. Furthermore, rehabilitation professionals, unless being a small group of participants, were particularly motivated to obtain their exam as 40.2 % were successful. We can hypothesize that most of them were physiotherapists as they belonged to the new group of NRT prescribers and are particularly concerned by caring smoking-related diseases in their everyday practice. Interestingly, we also identified professionals from the social sector, comprising around 3 % of the participants. Social workers are in an ideal position to assist individuals in SC, particularly disadvantaged smokers. They frequently interact with populations more likely to smoke and can build trust by assisting with various everyday life issues [6].

In our study, we achieved a higher success rate than that reported in the literature (15.9 % on FUN and 19.3 % on PNS) as completion rates for MOOCs typically fall below 13 % [7]. This result might be related to the incorporation of practical workshops with real-life examples in the MOOC content that enhance participant engagement and reinforce the applicability of acquired knowledge [8]. Moreover, hybrid teaching methods, including clinical cases explained by tobacco specialists or through patient testimonials, would not only promote a better understanding of the issues and practices involved in SC, but also provide a real dimension to learning, which is essential for training competent and empathetic professionals.

The proficiency of healthcare professionals is vital for delivering effective smoking cessation interventions [9]. Notably, MOOCs may help overcome barriers to providing adequate care for individuals struggling with SC. Indeed, it appears that teaching health professionals how to help individuals quit smoking is not very effective in the formal curriculum [10]. In the MOOC content, the emotional support provided through the workshops, which reflected empathy and supportive relationships, may have contributed to the development of health professionals' self-confidence in their ability to help individuals who smoke.

Our study results add knowledge on the topic of virtual training programs in SC with the inclusion of different types of healthcare professionals in the audience, extending the potential impact of the MOOC across disciplines. Despite the valuable insights gained, this study has limitations. Selection bias may have affected the study, notably the acceptance of questionnaire completion. Additionally, there were missing data among participants, as encountered in all survey-based studies, which may be attributed to non-response.

This study stands out in the realm of SC education, offering valuable insights into participant dynamics, engagement levels, and the overall effectiveness of a comprehensive MOOC targeting healthcare professionals. In fact, MOOCs provide a scalable and accessible method for equipping healthcare professionals, with essential SC skills, potentially serving as a model for future public health initiatives aimed at improving tobacco control. Policymakers should integrate evidence-based SC training into professional development programs for healthcare workers, prioritizing expanded access to training resources via digital platforms.

## Authors’ contributions

DA, YCB, and ALLF conceived and designed the study. DA and YCB were responsible for data curation, formal analysis, methodology, software, validation, and the original draft of the manuscript. IA, MM, AD, and ALLF contributed to project funding, management and writing – review and editing. MM and IA also contributed to investigation. All authors approved the final manuscript for submission.

## Ethical statement

This study did not require ethical approval, as the used platforms France Université Numérique (FUN) and Pédagogie Numérique en Santé (PNS) provide pseudonymized information on access, use, impact, and performance of learners. According to the Helsinki declaration, informed consent was gathered from persons who were filmed for the MOOC videos.

## Funding

Funding for the diffusion and evaluation of the MOOC “Smoking: Quit Your Own Way!” was provided by the Fonds de Lutte Contre les Addictions (Appel A Projet de la société civile 2019) through the Société Francophone de Tabacologie. The MOOC conception was funded by the Agence Nationale pour la Recherche (15-IDFN-0003) as part of the MOOCLIVE project.

## Declaration of competing interest

The authors have no conflict of interest to declare.

## Data Availability

The data that support the findings of this study are available from the corresponding author, upon reasonable request.
