# Supplementary material for: Empowering healthcare professionals to help smokers quit: Relevance of a smoking cessation online training program
Source: Public Health Pract (Oxf). 2025 Dec 11;11:100699. doi: 10.1016/j.puhip.2025.100699 (PMC12767846; doi:10.1016/j.puhip.2025.100699)
Supplement: Multimedia component 1 [file mmc1.docx]

**Supplemental materials**

**Methods**

*MOOC Description*

Practical workshops, recorded with individuals attempting to quit smoking in Georges Pompidou University Hospital in Paris, were also included to illustrate scientific information. The smokers participating in these workshops were receiving social benefits for unemployment, disability, invalidity, or minimum income at the time the videos were recorded. These workshops addressed topics such as the health consequences of smoking, misconceptions on tobacco use and smoking cessation medications, budgeting, and stress management tools. During these workshops, participants were encouraged to openly express their fears, motivations, and preconceived notions about quitting smoking, notably concerning the use of smoking cessation medications and/or electronic cigarettes. Game-based interventions were integrated to build practical skills while fostering a supportive social environment where participants could exchange personal strategies for avoiding smoking. While these workshops originally lasted 1.5 hours, they were condensed into 10-minute MOOC videos and supplemented with practical tools, including game guides and simplified scientific explanations tailored to participants' literacy levels.

*Data collection*

Participants' professions were grouped into 11 categories, with some professions being consolidated. For instance, nurses and advanced practice nurses, as well as general practitioners and other specialist doctors, have been merged into the broader categories of nurses and medical doctors, respectively. Additionally, physiotherapists, psychomotor therapists, speech therapists, and dieticians have been grouped under the category of “Rehabilitation professionals”.

**Results**

Figure S1 investigates the quiz success rates according to the weekly modules and the platforms. Beginning with the quizzes of week 1, 20.7% of learners successfully completed them on FUN and 30.0% on PNS, in contrast to the respective 4.3% and 2.2% who did not. A significant portion, 75.0% and 67.9%, did not participate in the quizzes of week 1, consequently being non-responders. As the course progressed, the weekly quiz success rates consistently declined on the PNS platform. On the FUN platform, the weekly quiz success rates declined from 18.1% in week 2 to 15.5% in week 5. The success rate then increased in the quizzes of weeks 6 and 7, reaching 28.2% and 28.5%, respectively.

**Table S1.** Weekly modules of the 7-week Massive Open Online Course (MOOC) "Smoking: Quit Your Own Way!"

| **Course modules** | **Components** |
| --- | --- |
| **1. Tobacco and health** | Metabolic diseases and diabetes |
|  | Chronic obstructive pulmonary disease (COPD) |
|  | Skin and teeth |
|  | Women's heart: specific effects of smoking and cessation |
|  | Psychiatric diseases: smoking prevalence, depression and suicidality risks, smoking cessation |
|  | Workshop on the health consequences of smoking |
| **2. Dependence** | Definition of addiction |
|  | Vulnerability to smoking: Contribution of nicotinic acetylcholine receptors |
|  | Story of a young smoker |
|  | Light smokers: health risks and smoking cessation success |
|  | Reduced tobacco consumption and risks: Use of heated tobacco compared with smoked tobacco |
|  | Addiction hospital wards: an opportunity to give up smoking |
|  | Relapse prevention groups: An example from Germany |
| **3. Who can help smokers to quit?** | Motivational interviews in pharmacies |
|  | Hospital bedside counseling team |
|  | Weight control and dietetics when quitting smoking |
|  | Role of the physiotherapist in tobacco cessation |
|  | Pregnant women who smoke: Managing cessation during pregnancy |
|  | Chronic Obstructive Pulmonary Disease: role of lung specialists and care providers |
| **4. How can we organize help for smokers?** | The Tab'agir network: A primary care network of health professionals trained in smoking cessation (Burgundy region – France) |
|  | The TABADO support school program: Helping adolescents to quit |
|  | Smoking cessation provided by Health Prevention university departments for university students |
|  | Smoking prevention in the workplace: how to help employed persons to quit? |
|  | League Against Cancer: the social inequalities in cancer prevention |
|  | Smoke-free month: A marketing social campaign since 2016 |
|  | National quitlines: Tabac Info Service (TIS) phone assistance and eTIS, the French national website to quit |
| **5. Tools to help smokers in their first quit attempt** | Motivational interviewing in smoking cessation: key-points |
|  | Workshop on the misconceptions on tobacco use and tobacco-related risks |
|  | Stress management workshop |
|  | Tobacco cessation and budget workshop |
|  | Optimal use of validated treatments in smoking cessation and electronic cigarettes to stop smoking |
|  | Workshop on the use of smoking cessation treatments |
| **6. Smoking cessation and tobacco control in Europe** | The French Interministerial Mission for Combating Drugs and Addictive Behaviors (MILDECA) |
|  | Epidemiology of smoking in France: Trends, tobacco-related social inequalities in health and effectiveness of public health measures |
|  | Health insurance coverage for smoking cessation treatments |
|  | Tobacco control in France |
|  | Tobacco control in Belgium |
|  | Tobacco control in Spain |
|  | Tobacco control in Switzerland: The example of smoking cessation for disadvantaged smokers |
| **7. The fight against tobacco beyond the borders of continental Europe** | The World Health Organization (WHO)'s role in the fight against smoking: the MPOWER program |
|  | WHO Framework Convention on Tobacco Control |
|  | The situation of smoking in Australia among low-income smokers |
|  | Smoking cessation in the United Kingdom: the best ways to quit smoking |
|  | The tobacco epidemic in Africa |

**Table S2.** Characteristics of the participants on both the France Université Numérique (FUN) and the Pédagogie Numérique en Santé (PNS) platforms

|  | **FUN** | **PNS** |
| --- | --- | --- |
| **Gender** | ***N=4,223*** | ***N=1,443*** |
| Female | 3,365 (79.7) | 1,212 (84.0) |
| Male | 836 (19.8) | 231 (16.0) |
| Other/Not specified | 22 (0.5) | 0 |
| **Age** | ***N=3,825*** | ***N=1,456*** |
| Median (range) | 34 (26–44) | 36 (28–47) |
| **Country of residence** | ***N=4,223*** | ***N=1,484*** |
| France | 3,688 (87.3) | 1,319 (88.9) |
| French-speaking countries | 427 (10.1) | 118 (7.9) |
| Non-French-speaking countries | 108 (2.6) | 47 (3.2) |
| **Educational level** | ***N=4,217*** | ***N=1,484*** |
| Secondary school diploma | 12 (0.3) | 8 (0.5) |
| Bachelor's degree | 396 (9.4) | 51 (3.4) |
| Postgraduate degree | 3,440 (81.6) | 1,368 (92.2) |
| Other | 369 (8.8) | 57 (3.8) |
| **Work sector** | ***N=1,758*** | ***N=1,482*** |
| Healthcare sector | 1,010 (57.5) | 1,029 (69.4) |
| Public administration | 96 (5.5) | 29 (2.0) |
| Private company sector | 119 (6.8) | 32 (2.2) |
| Education/research | 53 (3.0) | 17 (1.1) |
| Social protection (health insurance sector, social workers, etc.) | 21 (1.2) | 25 (1.7) |
| Unemployed | 289 (16.4) | 26 (1.8) |
| Voluntary/non-governmental sector | 64 (3.6) | 64 (4.3) |
| Other | 106 (6.0) | 260 (17.5) |
| **Type of professionals** | ***N=1,752*** | ***N=1,440*** |
| Nurses | 485 (27.7) | 412 (28.6) |
| Midwives | 55 (3.1) | 302 (21.0) |
| Medical doctors | 247 (14.1) | 284 (19.7) |
| Rehabilitation professionals | 94 (5.4) | 105 (7.3) |
| Psychologists | 83 (4.7) | 75 (5.2) |
| Pharmacists | 26 (1.5) | 49 (3.4) |
| Prevention professionals | 61 (3.5) | 44 (3.1) |
| Social professions | 59 (3.4) | 32 (2.2) |
| Dentists | 45 (2.6) | 19 (1.3) |
| Nurse assistants | 16 (0.9) | 14 (1.0) |
| Others (health students, laboratory technicians, teachers) | 581 (33.2) | 104 (7.2) |

Data are expressed as n (%), unless otherwise specified.

**
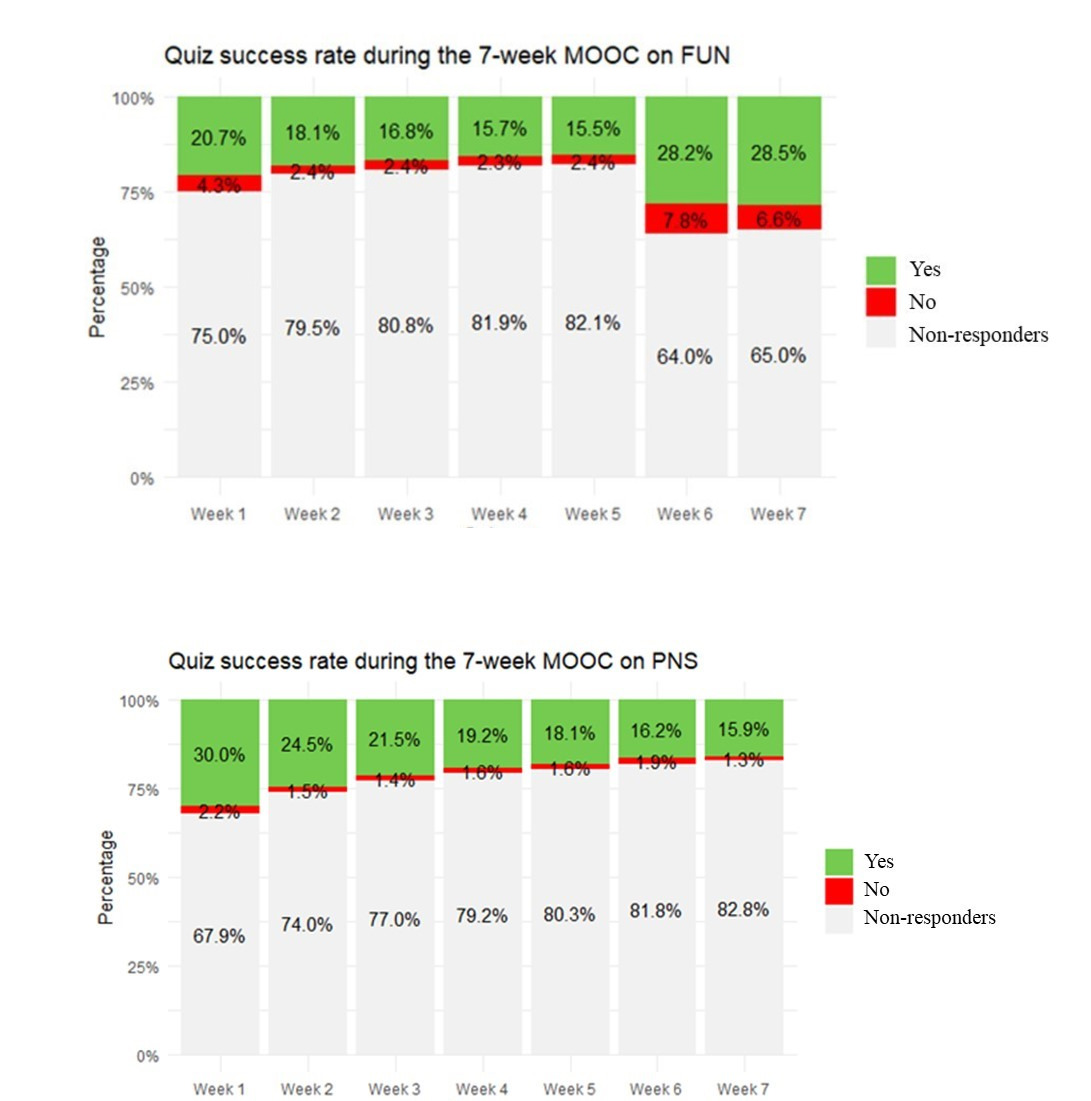
**

**Figure S1.** Quiz success and dropout rates during the 7-week Massive Open Online Course (MOOC), entitled "Smoking: Quit Your Own Way!", offered on the France Université Numérique (FUN) and the Pédagogie Numérique en Santé (PNS) platforms.


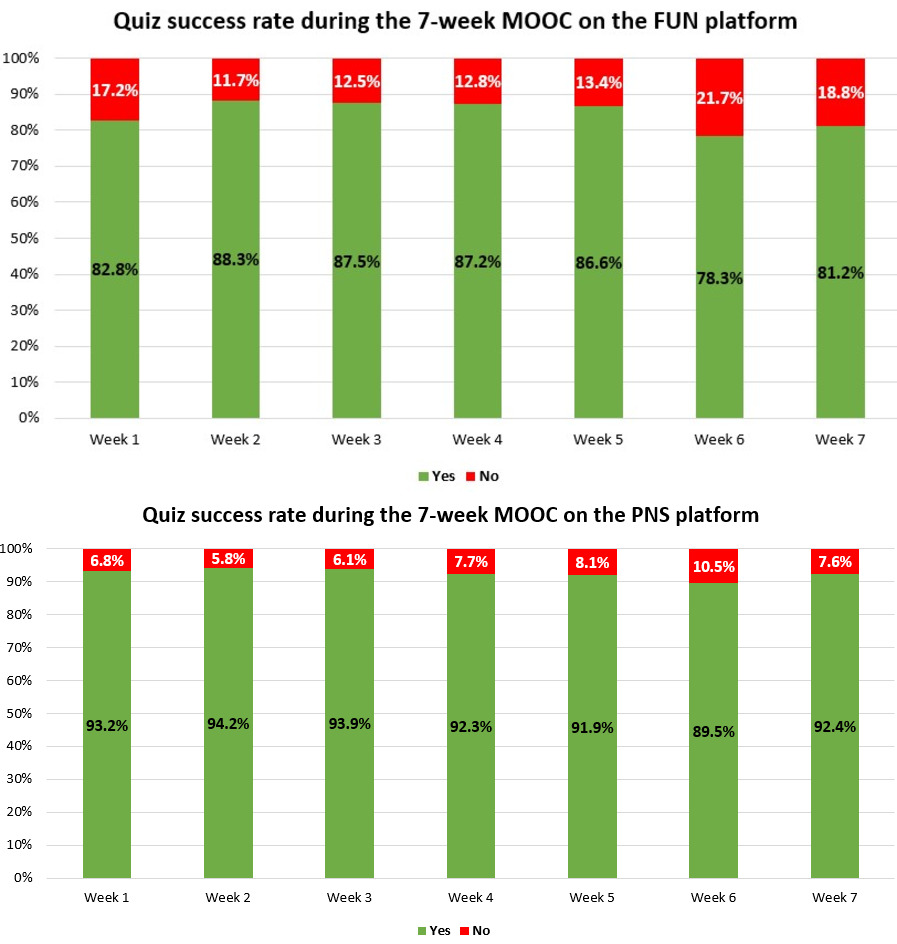


**Figure S2.** Quiz success rates, when excluding non-responders, during the 7-week Massive Open Online Course (MOOC), entitled "Smoking: Quit Your Own Way!", offered on the France Université Numérique (FUN) and the Pédagogie Numérique en Santé (PNS) platforms.
